# 自身抗体预后标志物在肿瘤免疫检查点抑制剂治疗中的临床价值

**DOI:** 10.3779/j.issn.1009-3419.2022.101.28

**Published:** 2022-07-20

**Authors:** 丽源 代, 晓红 韩

**Affiliations:** 1 100021 北京，国家癌症中心，国家肿瘤临床医学研究中心，中国医学科学院北京协和医学院肿瘤医院检验科 Department of Clinical Laboratory, National Cancer Center/National Clinical Research Center for Cancer/Cancer Hospital, Chinese Academy of Medical Sciences & Peking Union Medical College, Beijing 100021, China; 2 100730 北京，中国医学科学院北京协和医院临床药理研究中心；疑难重症及罕见病国家重点实验室；国家药监局药物临床研究与评价重点实验室；创新药物临床PK/PD北京市重点实验室 Clinical Pharmacology Research Center, Peking Union Medical College Hospital, State Key Laboratory of Complex Severe and Rare Diseases, NMPA Key Laboratory for Clinical Research and Evaluation of Drug, Beijing Key Laboratory of Clinical PK & PD Investigation for Innovative Drugs, Chinese Academy of Medical Sciences & Peking Union Medical College, Beijing 100730, China

**Keywords:** 免疫检查点抑制剂治疗, irAE, 自身抗体, 预后标志物, Immune checkpoint therapy, IrAE, Autoantibody, Prognostic marker

## Abstract

血清自身抗体标志物具有标本易获取、检测技术简便、可实现实时动态监测等优势。随着免疫检查点抑制剂在肿瘤治疗中的广泛应用，自身抗体标志物在肿瘤免疫检查点抑制剂治疗疗效、预后及免疫相关不良事件（immune related adverse events, irAEs）发生预测方面的报道逐渐增多，显示出了良好的预测潜力。本文主要探讨自身抗体标志物在肿瘤免疫治疗疗效、预后及irAE预测方面的研究进展，并对未来前景做出展望。

## Abstract (en)

Serum autoantibody markers have the advantages of easy specimen acquisition, simple detection technology and dynamic real-time monitoring. With the wide application of immune checkpoint inhibitors in the treatment of malignant tumors, autoantibody markers in predicting tumor immune checkpoint inhibitors efficacy and forecasting irAEs (immune related adverse events) show good prediction of potential. This review mainly focused on the progress of autoantibody markers in the prediction of therapeutic effect and the monitoring of irAE in tumor immunotherapy.

恶性肿瘤是危害人类健康的一大类疾病，针对抗程序性死亡受体1/程序性死亡配体1（programmed cell death-1/ programmed cell death ligand-1, PD-1/PD-L1）、细胞毒性T淋巴细胞相关蛋白4（cytotoxic T-lymphocyte associated protein 4, CTLA-4）等免疫检查点抑制剂（immune checkpoint inhibitors, ICIs）在多瘤种中的相继应用，揭开了肿瘤免疫治疗新篇章。免疫治疗显著地改善了肿瘤患者的总生存期（overall survival, OS）。然而，免疫单药治疗在绝大多数未经挑选的实体瘤中客观缓解率低（10%-30%）^[1]^、免疫相关不良事件（immune related adverse event, irAE）发生等问题日益引起人们的关注^[2]^。现有免疫治疗疗效预测标志物：肿瘤细胞表面PD-L1表达水平、肿瘤突变负荷（tumor mutational burden, TMB）、微卫星不稳定性/错配修复缺陷（microsatellite instability-high/deficient mismatch repair, MSI-H/dMMR）^[3-5]^等，存在如下问题：PD-L1表达检测抗体多样，检测设定阈值不统一^[6]^；MSI-H/dMMR在人群中发生率仅为1%-25%^[7]^；TMB检测价格较为昂贵，结果更加依赖组织标本质量，阈值不统一。因此，积极探索新的免疫治疗预后标志物是有必要的。

随着人们对肿瘤早诊早治及精准医疗的追求，血清自身抗体标本易获得、易于检测、长半衰期、高特异性、非侵袭性等优势逐渐凸显。自身抗体在肿瘤早期诊断方面已经显示出了巨大的应用优势及前景，其用于预测ICIs治疗疗效、预后及irAE的研究报道也在逐渐增多。本文着重探讨自身抗体标志物在预测免疫治疗疗效、预后及irAE发生两大方面的研究进展。

## 1 肿瘤相关自身抗体

肿瘤相关抗原（tumor associated antigen, TAA）指在肿瘤细胞或正常细胞上存在的抗原分子。在肿瘤发生发展的极早期，肿瘤细胞就能表达TAA被人体免疫系统所识别，产生肿瘤相关自身抗体（tumor associated autoantibody, TAAb）^[8]^。目前已知TAA产生机制包括：机体免疫耐受缺陷使得自身反应性B细胞重获反应、蛋白质表达水平或结构改变、肿瘤细胞死亡等导致TAA过表达等^[9]^。检测自身抗体的常用技术包括：血清学筛选互补DNA表达文库、间接酶联免疫吸附实验及高通量蛋白质芯片等。

血清自身抗体在肿瘤诊断方面的应用已有广泛研究。在肺癌及乳腺癌诊断方面，已有上市试剂盒：肺癌Early-CDT^TM^（美国）、肺癌7种自身抗体诊断试剂盒（中国）、乳腺癌Videssa Breast试剂盒等^[10-12]^。在肿瘤治疗预后预测方面，血清自身抗体标志物在非小细胞肺癌（non-small cell lung cancer, NSCLC）^[13]^、乳腺癌^[14]^、肝癌^[15]^、结直肠癌^[16]^、食管鳞状细胞癌^[17]^等多个瘤种中均有报道。其中，纳入多种治疗方式（含手术治疗、化疗、放疗等）的研究占绝大多数，纳入单一治疗方式中手术治疗后的预测作用探索较多。研究最多的自身抗体为p53自身抗体，p53自身抗体阳性与患者不良预后密切相关。例如在非小细胞肺癌NSCLC手术治疗中，p53自身抗体被发现可预测NSCLC患者中位无进展生存期（progression-free survival, PFS）及OS，自身抗体水平越高，预后越差^[13]^。其他自身抗体在肿瘤预后预测中的研究包括：抗骨桥蛋白（osteopontin, OPN）自身抗体在肝癌中与不良预后相关^[15]^、抗人类DNA拓扑异构酶Ⅰ（human DNA-topoiomerase Ⅰ, TOPO48）自身抗体在食管鳞状细胞癌中水平与良好预后相关等。提示自身抗体滴度与肿瘤复发状态密切相关，肿瘤复发产生的抗原刺激机体免疫系统使得血清自身抗体水平升高，能够起到复发监测的作用^[18]^。

血清自身抗体在肿瘤免疫治疗疗效、预后及irAE预测方面的报道日益增多。由免疫反应所识别的TAA，是阐明癌症发展及药物反应分子机制的方法^[18-21]^。此外，肿瘤组织抗原谱和任何预先存在的对肿瘤抗原的免疫反应可能有助于预测肿瘤治疗（化疗、靶向治疗、免疫治疗等）反应。例如：在弥漫大B细胞淋巴瘤及套细胞淋巴瘤行含利妥昔单抗靶向联合化疗的R-CHOP治疗患者中，分别发现基线血高水平的亨廷顿相互作用相关蛋白1（huntingtin interacting protein 1-related, HIP1R）自身抗体、低密度脂蛋白受体相关蛋白（low-density lipoprotein receptor-related protein-associated protein 1, LRPAP1）自身抗体与更好的免疫治疗预后（PFS及OS）相关^[19, 22]^。提示自身抗体水平与抗原表达及机体免疫功能状态有关，能够较好地反映机体免疫功能状态。而机体免疫功能状态与ICIs疗效紧密相关，因而自身抗体具有作为ICIs治疗疗效及预后标志物的潜能。自身抗体标志物用于肿瘤ICIs治疗疗效、预后及irAE预测方面的研究总结见表 1和表 2。

**表 1:** 自身抗体标志物用于肿瘤ICIs治疗疗效及预后预测 Autoantibody markers for therapeutic and prognosis efficacy in ICIs therapy

Treatment	Cancer type	Sample size	Autoantibodies	Prognostic endpoint	Ref.
CTLA4 monoclonal antibody	Melanoma	15	NY-ESO-1 Ab	PD/Non-PD	[26]
		144	NY-ESO-1 Ab	PD/Non-PD	[27]
		41	NY-ESO-1, gp100, MelanA/MART1, TRP1/TYRP1, TRP2/TYPR2 Ab	PD/Non-PD OS	[29]
	SCLC	38	SOX2, anti-Hu, anti-Yo, VGCC, VGPCA, ANA, ANCA Ab	PD/Non-PD	[31]
		38	HuD, Gad65, Sox1, Ma1, Ma2, Amphiphysin, CRMP5, Ri, Yo Ab	PFS, OS	[32]
PD-1 monoclonal antibody	NSCLC	137	ANA, thyroglobulin, thyroid peroxidase Ab	PFS	[33]
		42	lgM-RF	PD/Non-PD PFS, OS	[34]
		88	NY-ESO-1, XAGE1 Ab	PFS, OS	[35]
		166	NY-ESO-1, p53, BRCA2, HUD, TRIM21 Ab	ORR, PFS	[37]
	NSCLC, ASPS, Lymphoma	74	SIX2, EIF4E2 Ab	PD/Non-PD	[38]
CTLA4: cytotoxic T-lymphocyte associated protein 4; PD-1: programmed cell death 1; NSCLC: non-small cell lung cancer; SCLC: small cell lung cancer; ASPS: alveolar soft part sarcoma; NY-ESO-1: new york esophageal squamous cell carcinoma 1; gp100: glycoprotein; MelanA/MART1: melanoma antigen; TRP1/TYRP1; TRP2/TYPR2: melanoma cell differentiation antigen tyrosinase-associated proteins 1 and 2; SOX2: SRY homeobox proteins; Hu: human protein antigen; Yo: purkinje cell cytoplasm type 1; VGCC: voltage-gated calcium channel antibody; VGPCA: antibody against voltage-gated potassium channel; ANA: antinuclear antibodies; ANCA: antineutrophil cytoplasmic antibody; HuD, Gad65, Sox1, Ma1, Ma2, Amphiphysin, CRMP5, Ri, Yo: neuro-associated autoantibodies; lgM-RF: immunoglobulin M-rheumatoid factors; XAGE1: tumor-testicular antigen; p53: p53 protein; BRCA2: BRCA2 protein; TRIM21: Tripartite motif containing-21; SIX2: homeobox protein SIX2; EIF4E2: eukaryotic translation initiation factor 4E type; PD/Non-PD: disease-progression/non-progression; PFS: progression-free survival; OS: overall survival; ORR: objective response rate.					

**表 2:** 自身抗体标志物用于肿瘤ICIs治疗irAE预测 Autoantibody markers for prediction of irAE in ICIs therapy

Treatment	Cancer type	Sample size	Autoantibodies	irAE	Ref.
CTLA4 monoclonal antibody	Melanoma	133	23 autoimmune disease-related autoantibodies	Arthritis, colitis, dermatitis and so on	[43]
PD-1/PD-L1 monoclonal antibody	NSCLC	40	BP180 Ab	Dermatitis (eg. itching, rash)	[46]
ICIs mono/combined therapy	Melanoma	78	SVM model	Gastroenteritis, dermatitis and so on	[47]
ICIs: immune checkpoint inhibitors; BP180: bullous pemphigoid antigen 180; PD-L1: programmed cell death ligand 1; irAE: immune related adverse event; SVM: support-vector-machine.					

## 2 肿瘤ICIs治疗疗效及预后预测相关自身抗体

### 2.1 CTLA-4单抗免疫治疗

CTLA-4单抗疗效及预后相关自身抗体主要研究集中于纽约食管鳞状细胞癌1（new york esophageal squamous cell carcinoma 1, NY-ESO-1）自身抗体，NY-ESO-1属于肿瘤睾丸抗原，最早于1997年在食管癌患者血清中被发现，在正常组织中不表达，在恶性肿瘤中高频率表达，已经在包括黑素瘤和肺癌、肝癌等多种肿瘤中被报道^[23]^。NY-ESO-1具有高度免疫原性，是有希望的免疫治疗靶点^[9]^。针对NY-ESO-1的肿瘤抗原疫苗临床试验已有陆续开展，并取得了一定的临床效果^[23]^。

NY-ESO-1在大约20%黑色素瘤患者中表达，在疾病晚期的频率更高，在癌症患者中NY-ESO-1可引发自发的体液和细胞反应^[23-25]^。Yuan等^[26]^最早在接受伊匹木单抗治疗的转移性黑色素瘤患者中被发现，基线血血清中NY-ESO-1自身抗体阳性患者更有可能获得临床获益。该研究结果在更大队列的样本（*n*=144）中得到了验证，提示NY-ESO-1自身抗体可能是晚期黑色素瘤患者CTLA-4单抗治疗疗效预测的重要指标^[27]^。Postow等^[28]^在病例报告中发现：在使用伊匹木单抗时，随着肿瘤负荷增大，NY-ESO-1血清抗体呈现缓慢上升趋势；加上放疗后，疾病控制，血清抗体下降。首次通过动态监测NY-ESO-1自身抗体水平预测了伊匹木单抗治疗疗效。而后，Fässler等^[29]^在PD-1单抗治疗中发现：包括NY-ESO-1在内的共5种肿瘤睾丸抗原及黑色素瘤细胞分化抗原诱导产生的抗体基线水平在免疫应答组中同样更高。

除此之外，自身抗体标志物预测CTLA-4单抗治疗小细胞肺癌（small cell carcinoma, SCLC）疗效及预后也有文献报道，已知在SCLC患者治疗过程中，经常伴随神经系统副肿瘤综合征，已有研究^[30]^证实anti-Hu等神经相关自身抗体（anti-neuronal autoantibodies, NAA）能够预测伊匹木单抗治疗SCLC疗效。Arriola等^[31]^在接受伊匹木单抗联合卡铂及依托泊苷的晚期SCLC患者中检测基线血血清中7种自身抗体，发现基线血自身抗体阳性患者PFS显著延长（8.8个月 *vs* 7.3个月）。此外，Hardy-Werbin等^[32]^对时序性（基线血及第一次疗效评价、疗效评价进展时）血清中NAA进行检测发现：单独化疗患者中，任一自身抗体阳性与更长的OS相关（15.1个月 *vs* 11.7个月）；免疫联合化疗患者中，OS也有明显的趋势（12.3个月 *vs* 7.9个月）。

现有CTLA-4单抗免疫治疗疗效及预后相关自身抗体标志物探索性研究主要集中在黑色素瘤及SCLC当中，基线血NY-ESO-1自身抗体阳性与更好的疗效及预后相关。此外，NY-ESO-1自身抗体不仅可用于CTLA-4单抗治疗疗效、预后预测及动态监测，还可预测PD-1单抗治疗疗效及预后，显示出良好的临床应用前景。

### 2.2 PD-1/PD-L1单抗免疫治疗

现有文献报道与PD-1/PD-L1单抗疗效及预后相关的自身抗体包括：①自身免疫性疾病相关抗体；②肿瘤睾丸抗原及肿瘤发生发展相关自身抗体；③新筛选发现（*de*-*novo*）自身抗体等。主要涉及NSCLC的免疫治疗疗效、预后预测和监测。其中，除类风湿因子免疫球蛋白M（immunoglobulin M-rheumatoid factors, IgM-RF）抗体及同源框蛋白（homeobox protein SIX2, SIX2）自身抗体水平与ICIs治疗不良预后相关外，其余自身抗体标志物均与ICIs疗效及预后呈正相关，即：基线血自身抗体阳性或水平越高，患者治疗疗效及预后越好。

在检测自身免疫性疾病相关自身抗体方面，Toi等^[33]^在行纳武利尤单抗或帕博丽珠单抗单药治疗晚期NSCLC患者基线血血清中检测免疫性疾病相关自身抗体：类风湿因子、抗核抗体、抗甲状腺球蛋白、抗甲状腺过氧化物酶自身抗体，判定任何一个自身抗体阳性即为阳性。发现基线血自身抗体阳性患者，PFS更长（6.5个月 *vs* 3.5个月）。此外，Ugolini等^[34]^对行纳武利尤单抗或帕博丽珠单抗单药治疗的转移性NSCLC患者检测基线及动态IgM-RF水平，发现基线高IgM-RF水平与3个月内治疗进展、更短的PFS及OS正相关。通过机制研究证实IgM-RF通过减少发挥抗肿瘤T细胞作用的CD137^+^ T细胞，使PD-1单抗治疗产生初步耐药性。

在检测肿瘤睾丸抗原及肿瘤发生发展相关自身抗体方面，Ohue等^[35]^对接受PD-1单抗单药治疗的晚期NSCLC患者基线血血清进行NY-ESO-1、XAGE1（肿瘤睾丸抗原）自身抗体检测，发现自身抗体阳性患者客观缓解率更高（65% *vs* 19%, *P*=0.000, 6），多因素分析纳入肿瘤PD-L1表达进行校正，证实抗体阳性是PFS（HR=0.4, *P*=0.01）和OS（HR=0.2, *P*=0.004）的独立预测标志物。随后，Tarhoni等^[36]^检测晚期NSCLC患者基线血6个自身抗体，发现NY-ESO-1、波形蛋白（vimentin）等自身抗体差异性与OS呈显著负相关，与以往研究报道相悖，鉴于该研究报道纳入例数（*n*=40）较少，该研究结论尚待进一步验证。此外，Zhou等^[37]^在接受PD-1单抗单药或联合治疗晚期NSCLC患者基线血中发现包含p53自身抗体在内的5个自身抗体阳性与更好的客观缓解率（44.4% *vs* 13.6%, *P* < 0.001）及更长的PFS相关（7.6个月 *vs* 3.3个月，*P* < 0.001）。

以上研究多基于以往文献报道选定自身抗体检测，而针对人类蛋白质诱导机体产生的自身抗体进行广筛，实现新筛选发现（*de*-*novo*），多阶段逐步验证，从而筛选预测标志物的方法也有研究报道。Tan等^[38]^通过核酸蛋白微阵列（nucleic acid programmable protein arrays, NAPPA）高通量蛋白质芯片检测腺泡软组织肉瘤、NSCLC和淋巴瘤患者基线血血清中自身抗体水平。以6个月是否发生病情进展划分反应者和无反应者，发现在NSCLC中，SIX2自身抗体可有效区分反应者，受试者工作特征曲线下面积（area under the curve, AUC）达0.87，SIX2自身抗体水平越高，免疫治疗疗效越差；在淋巴瘤中，真核翻译起始因子4E2（eukaryotic translation initiation factor 4E type 2, EIF4E2）自身抗体AUC为0.7，EIF4E2自身抗体水平越高，免疫治疗疗效越好。

## 3 肿瘤irAE预测相关自身抗体

ICIs治疗通过解除对T细胞产生抑制的负性共刺激信号从而发挥增强抗肿瘤T细胞反应的作用。由于这种作用方式是非肿瘤抗原特异性的，在此过程中很有可能激活自身反应性T细胞，使得T细胞对肿瘤抗原及自身抗原的免疫耐受性中断，从而激活自身反应性B细胞，产生自身抗体。因此自身抗体的产生可能与irAE发生及机体全身免疫系统增强相关^[38-41]^。Osorio等^[42]^纳入Keynote001研究行帕博丽珠单抗单药治疗的晚期NSCLC共51例患者，发现irAE患者甲状腺功能障碍出现较早（中位数：42 d），有甲状腺功能障碍的患者OS更长（HR=0.29, 95%CI: 0.09-0.94, *P*=0.04）。

研究者^[43]^通过检测接受伊匹木单抗治疗的133例晚期黑色素瘤患者治疗前后血清中临床上常见的23种自身抗体（抗甲状腺自身抗体等）发现，疗后自身抗体阳性与irAE发生、更好的客观缓解率和OS呈显著正相关（HR=0.66, 95%CI: 0.34-1.26）。Kurimoto、Les等^[44, 45]^分别在多种晚期接受ICIs治疗的肿瘤患者（黑色素瘤、NSCLC、肾癌、胃癌等）中，同样发现基线血甲状腺自身抗体水平与甲状腺irAE发生显著正相关。提示预先存在的甲状腺自身免疫可能与ICIs治疗过程中irAE发生发展正相关，具有预测irAE的潜力。

在筛选irAE相关自身抗体研究方面，Hasan等^[46]^提出正常皮肤组织与NSCLC共有抗原是皮疹等irAE发生原因之一。通过数据库筛选NSCLC组织与正常皮肤组织分子组织指纹图谱RNA测序共有的10个基因，包括：大疱类天疱疮抗原180（bullous pemphigoid antigen 180, BP180）、BP230、Ⅶ型胶原蛋白等，发现接受PD-1/PD-L1单抗单药治疗的NSCLC患者中，根据3个月内病情是否进展界定ICIs治疗反应，基线血BP180自身抗体水平与皮肤irAE正相关（*P*=0.04），与更好的治疗反应（*P*=0.01）及OS（*P*=0.04）相关。Gowen等^[47]^使用HuProt人类蛋白质芯片分别分析接受CTLA4单抗、PD-1单抗或联合治疗的晚期黑色素瘤患者基线血自身抗体水平与irAE（皮肤毒性、甲状腺功能障碍等）的关系，建立了支持向量机（support-vector-machine, SVM）模型，通过识别基线血抗体特征预测irAE。SVM模型区分irAE组与无irAE组的准确率、敏感性和特异性均大于90%。与该研究类似的de-novo设计同样发现早期监测到的自身抗体与irAE发生呈正相关^[48]^。

## 4 小结与展望

随着ICIs在肿瘤治疗中的广泛应用，寻找预测、监测ICIs治疗疗效及预后有效且实用的生物标志物日益受到重视。现有肿瘤组织PD-L1检测、TMB、MSI-H/dMMR存在检测平台及方法流程、标准尚未统一化、组织标本难以获取、难以实现动态监测等问题。肿瘤相关自身抗体已在肿瘤诊断当中取得了进展，并具有样本易获取、检测技术简便、可实现实时动态监测等优势，在预测黑色素瘤及肺癌CTLA4、PD-1单抗治疗疗效、预后及irAE方面显示出了较好的预测潜能。

目前，肿瘤自身抗体标志物在免疫治疗疗效、预后预测方面尚未在临床推广应用，分析可能原因为：①样本量小，瘤种单一、缺乏新的验证队列。现有研究瘤种多集中在黑色素瘤及晚期NSCLC，样本量纳入集中在50例左右，并且未在新队列中进一步确证；②纳入免疫联合治疗少。已有研究证实免疫联合治疗（联合化疗/抗血管治疗）疗效优于免疫单药，现有探索大多仅纳入单药治疗。此外，自身抗体标志物在化疗或抗血管治疗方式中的探索研究同样较少；③*de*-*novo*研究占比少，缺乏机制探索。现有文献多基于已有文献报道自身抗体检测，且未进行机制探索；④缺乏其他预测因素校正。未纳入PD-L1/TMB等进行比较或联合分析；⑤未联合多指标建模预测。相对单一指标进行疗效、预后预测，联合蛋白质标志物等，可提高预测灵敏度，未来应联合多指标多组学数据构建预测模型；⑥未进行疗效实时动态监测探索。现有文献多基于基线血预测免疫治疗疗效及预后，未来应充分发挥TAAb易于取样、检测简易等优势，动态监测实时反映治疗效果。

综上所述，肿瘤相关自身抗体相对于现有疗效预测标志物有着独特的优势，但由于上述原因的存在，尚无成功转化至临床应用的先例。除纳入更大样本、在更多瘤种中进行队列验证、探索免疫联合治疗预测标志物、多指标多组学构建预测模型及实时动态监测外，还应注重自身抗体标志物的机制研究，这将在推动免疫治疗自身抗体标志物的发现及合理应用ICIs治疗的同时，有利于识别发现癌症发生发展机制、药物反应分子机制及开发新的免疫治疗靶点。
